# Circ_000829 Plays an Anticancer Role in Renal Cell Carcinoma by Suppressing SRSF1-Mediated Alternative Splicing of SLC39A14

**DOI:** 10.1155/2022/8645830

**Published:** 2022-08-26

**Authors:** Jia-fu Feng, Wen-yu Yang, Yao-dong Wang, Gang Xie, Bei Xu, Chun-mei Dai, Bin Zhang, Xiao-han Li, Jun Wang, Yu-wei Yang

**Affiliations:** ^1^NHC Key Laboratory of Nuclear Technology Medical Transformation, Mianyang Central Hospital, School of Medicine, University of Electronic Science and Technology of China, Mianyang 621000, China; ^2^Departments of Clinical Laboratory, Mianyang Central Hospital, School of Medicine, University of Electronic Science and Technology of China, Mianyang 621000, China; ^3^College of Medical Technology, Chengdu University of Traditional Chinese Medicine, Chengdu 611137, China; ^4^Departments of Urology Surgery, Mianyang Central Hospital, School of Medicine, University of Electronic Science and Technology of China, Mianyang 621000, China; ^5^Departments of Pathology, Mianyang Central Hospital, School of Medicine, University of Electronic Science and Technology of China, Mianyang 621000, China; ^6^Department of Medical Laboratory, Affiliated Hospital of Southwest Medical University, Luzhou 646000, China

## Abstract

**Background:**

Covalently closed circular RNAs (circRNAs) play critical oncogenic or anticancer roles in various cancers including renal cell carcinoma (RCC), pointing to their regulation as a promising strategy against development of RCC. We, thus, studied the tumor-suppressive role of circ_000829 in RCC through *in vitro* and *in vivo* experiments.

**Methods:**

The expression of circ_000829 was validated in clinical RCC tissues and RCC cell lines. Based on ectopic expression and knockdown experiments, we examined the interactions among circ_000829, serine and arginine rich splicing factor 1 (SRSF1), and solute carrier family 39 member 14 (SLC39A14, zinc transporter). Then, the effects of circ_000829, SRSF1, and SLC39A14 on cell cycle distribution and proliferation *in vitro* and on tumor growth *in vivo* were evaluated in RCC cells.

**Results:**

Circ_000829 was poorly expressed in RCC tissues and cells, while SRSF1 was highly expressed. Restoration of circ_000829 reduced the levels of SRSF1 and SLC39A14B, thereby repressing the RCC cell proliferation *in vitro* and tumor growth *in vivo*. Meanwhile, overexpression of SRSF1 and SLC39A14B promoted the proliferation and cell cycle entry of RCC cells. Mechanistically, circ_000829 directly bound to SRSF1, and SRSF1 enhanced the expression of SLC39A14B by mediating the alternative splicing of SLC39A14. SLC39A14B upregulation negated the effect of SLC39A14 knockdown on RCC cell proliferation.

**Conclusion:**

Hence, this study suggests the antiproliferative role of circ_000829 in RCC growth and further elucidates the underlying mechanism involving the inhibited SRSF1-mediated alternative splicing of *SLC39A14* mRNA.

## 1. Introduction

Renal cell carcinoma (RCC) originates from the epithelial cells of the renal tubules [1, 2]. Previous evidence has shown that the combination of cabozantinib, nivolumab/ipilimumab, and atezolizumab/bevacizumab yields better efficacy than other treatments in the clinical treatment of RCC [3], but the three all have disadvantages including narrow range of use, large differences in efficacy, or high treatment costs. At present, in-depth study of the carcinogenic mechanism of RCC has led to the clinical approval of several therapeutic drugs targeting vascular endothelial growth factor or mTOR pathway. However, the therapeutic responses in patients with advanced RCC are still unsatisfactory [4], thus calling for still more effective targeted therapies.

Serine and arginine rich splicing factor 1 (SRSF1) is highly expressed in a variety of tumor cells and plays a role through alternative splicing of genes related to cancers [5]. Indeed, during development, over 95% human genes undergo alternative splicing, and the misregulation of alternative splicing often leads to diseases including cancers [6]. For instance, SRSF1 is involved in the alternative splicing of solute carrier family 39 member 14 (SLC39A14, zinc transporter) through the Wnt pathway in colorectal cancer cells [7]. SLC39A14, also known as ZIP14, has two alternatively spliced products, SLC39A14A (ZIP14A) and SLC39A14B (ZIP14B). Although there is no difference in the total expression of ZIP14, the ratio of ZIP14A to ZIP14B in tumor cells may also be significantly lower than that in normal tissues. Therefore, ZIP14 splicing variant was differentially expressed in cancer tissues [8]. Moreover, evidence has been presented demonstrating the involvement of SRSF1 in the malignant biological processes of RCC cells, which may indicate unfavorable survival RCC patients [9, 10]. However, the specific mechanisms of SRSF1 in RCC still need to be further studied.

Circular RNAs (circRNAs) are regulators of various biological mechanisms that can inhibit tumorigenesis [7, 11]. There is a paucity of data have confirmed the regulatory effect of circRNAs in the development of RCC, pointing to the importance of circRNAs in developing new targets for RCC therapy [12–14]. Of note, evidence exists indicating that circRNAs modulate alternate splicing of mRNAs by binding to SRSF1. For instance, circRPAP2 was suggested to bind to SRSF1 and subsequently repressed SRSF1-mediated alternate splicing of PTK2, resulting in the suppression of breast carcinogenesis [15]. However, the mechanistic actions between circRNAs and SRSF1 in RCC have been poorly explored. Herein, we first determined the abnormal expression of SRSF1-related circRNAs in RCC by bioinformatics analysis and identified circ_000829 as a key circRNA that might bind to SRSF1. Then, we explored the role of circ_000829 in RCC and elaborated the underlying mechanisms.

## 2. Material and Methods

### 2.1. Ethics Statement

The current study was approved by the Ethics Committee of Mianyang Central Hospital (S2018085, P2020030) and performed in strict accordance with the Declaration of Helsinki. All participants or legal guardians had signed the informed consent documentation prior to sample collection. Animal experiments were approved by the Animal Ethics Committee of Mianyang Central Hospital (P2020030).

### 2.2. Bioinformatics Analysis

The expression matrix of the RCC-related circRNA microarray dataset GSE100186 and its comment probe file was downloaded from the Gene Expression Omnibus database. Differentially expressed circRNAs were screened from the GSE100186 dataset (|logFC| > 2, *p* < 0.05) using the R language “limma” package. GSE100186 included four RCC samples and four normal samples. A circRNA-RNA binding protein interaction analysis was performed to predict the circRNAs that likely interact with SRSF1 through the Circinteractome website. The expression of SLC39A14B in RCC tissues and normal tissues was compared by the GEPIA site, an interactive web server of cancer expression profiles.

### 2.3. Study Cohort and Sample Collection

A total of 67 paired RCC tissues and adjacent normal tissues were obtained from RCC patients, including 43 males and 24 females aged 35-76 (56.7 ± 12.0) years, who underwent partial or radical nephrectomy with nephron preservation in the Urology Department of Mianyang Central Hospital from June 2017 to June 2019. The patients were pathologically diagnosed with RCC after surgery, but had not received preoperative neoadjuvant chemotherapy or immunotherapy [16]. The demographic and clinical characteristics are presented in Supplementary Table 1. The tissue samples were stored at -80°C for gene expression determination. The clinical samples were classified by two pathologists according to the WHO/ISUP classification. Grade I (13 cases) and grade II (26 cases) were further defined as the low-level group, and grade III (28 cases) was defined as the high-level group for analysis.

### 2.4. Northern Blot

As previously reported, digoxigenin-labeled antisense RNA probes (200 nt) were used [17]. Northern blot assay was performed using NorthernMax Kits (AM1940, Thermo Fisher, Waltham, MA) to evaluate the expression of circ_000829 in RCC tissues and adjacent normal tissues and that in A498 and 786-O cell lines in the circ_000829 knockdown presence. This assay was also employed to determine the SLC39A14 expression in RCC tissues and adjacent normal tissues and that in A498 and 786-O cell lines in response to SLC39A14 overexpression or knockdown. The blots were visualized using a ChemiDoc XRS+ imaging system (Bio-Rad, Hercules, CA) [18].

### 2.5. Immunohistochemical (IHC) Staining

Tissue specimens were paraffin-embedded and sectioned. The sections were treated with 3% methanol-H_2_O_2_ and then subjected to antigen retrieval. Next, the sections were blocked with normal goat serum and then incubated with primary rabbit antibodies (Abcam, Cambridge, UK) against SRSF1 (ab38017), Ki67 (ab15580), and UBE2C (ab252940) overnight at 4°C. The next day, the sections were reprobed with the goat anti-rabbit IgG (1 : 1000, ab6785, Abcam) at 37°C for 20 min and incubated with HRP-labeled streptavidin protein working solution. After DAB development and hematoxylin counterstaining, the images in five randomly selected high-power fields from each section were observed with a microscope. There were 67 samples in each group when IHC staining was performed to analyze the expression of SRSF1 in RCC tissues and adjacent normal tissues. There were 8 samples in each group when IHC staining was performed to analyze the expression of Ki67, SRSF1, and UBE2C in the tumors of nude mice.

### 2.6. Cell Culture

Human RCC A498 cell line (3111C0001CCC000171, Resource Center, Institute of Basic Medical Sciences, Academy of Medical Sciences, Beijing, China) was cultured with minimum essential medium containing 100 *μ*g/mL streptomycin, 100 U/mL penicillin, 10% FBS, and 1% NEAA. Human RCC 786-O cell line (111C0001CCC000243, Resource Center, Institute of Basic Medical Sciences, Academy of Medical Sciences) was cultured in RPMI-1640 supplemented with 100 *μ*g/mL streptomycin, 100 U/mL penicillin, and 10% FBS. The two cell lines were cultured in a 5% CO_2_ incubator at 37°C. Under cell confluency of approximately 80%, the cells were subcultured, and those at the third passage were selected for subsequent experimentation.

### 2.7. Cell Transduction

Cell suspension (200 *μ*L, 3 × 10^5^ cells/mL) at the third passage was plated onto a 96-well cell culture plate. Upon reaching 60-80% confluence, the A498 and 786-O cells were transduced using Lipofectamine 2000 reagent (Invitrogen Inc., Carlsbad, CA) with lentiviruses (Sino Biological Inc., Beijing, China) carrying short hairpin RNA (shRNA, sh-) targeting circ_000829 (labeled as sh-circ_000829), circ_000829 overexpression vector (labeled as circ_000829 vector), SRSF1 overexpression vector (labeled as SRSF1 vector), shRNA targeting SLC39A14 (labeled as sh-SLC39A14), and their corresponding negative controls (NCs: sh-NC or NC vector). Following 6 h of transduction, the medium was renewed, and the cells were then cultured for a further 48 h. Corresponding antibiotics were added to the cells to screen the stable cell lines for subsequent experiments.

### 2.8. Reverse Transcription-Quantitative Polymerase Chain Reaction (RT-qPCR)

Total RNA was extracted from cells and tissues using TRIzol reagent (15596-018, Solarbio, Beijing, China). The primers used were synthesized by Sangon (Shanghai, China) (Supplementary Table 2). RNA was reversely transcribed to complementary DNA using Reverse Transcription Kit (5081963001, Roche, Penzberg, Germany). Genes were amplified by RT-qPCR using a SYBR Green qPCR Master Mix kit (B21202, Bimake, Shanghai, China) on the Applied Biosystems ViiA™ 7 instrument (Life Technologies, Inc., Applied Biosystems, Foster City, CA). As normalized to *β*-actin, fold changes in gene expression were analyzed using the 2^-∆∆Ct^ method.

### 2.9. Western Blot

Total protein was extracted from tissues or cells using high-efficiency RIPA lysis buffer (R0010, Solarbio). The protein concentration was then determined using bicinchoninic acid kits (20201ES76, Yeasen, Shanghai, China). Protein samples were separated by sodium dodecyl sulfate-polyacrylamide gel electrophoresis and then electro-transferred onto a polyvinylidene fluoride membrane. The membrane was then blocked by 5% skimmed milk at room temperature for 1 h. After that, the membrane was incubated with primary antibodies against SLC39A14 (ab106568, 1 : 1000, Abcam), SRSF1 (ab38017, 1 : 1000, Abcam), and actin (ab8226, 1 : 5000, Abcam) at 4°C overnight. Next, the membrane was reprobed with diluted HRP-labeled secondary antibody of goat antirabbit IgG (ab205718, 1 : 20000, Abcam) and goat antimouse IgG (ab6789, 1 : 5000, Abcam) at room temperature for 1 h and then developed with enhanced chemiluminescence. The bands in western blot images were quantified by Image J analysis software, and the relative expression level was normalized to GAPDH.

### 2.10. Flow Cytometric Detection of Cell Cycle Distribution and Apoptosis

Cell suspension (200 *μ*L, 3 × 10^5^ cells/mL) at the third passage was plated onto a 96-well cell culture plate. PBS-rinsed cells were detached with trypsin and then centrifuged at 800 g to remove the supernatant. The pelleted cells were fixed with 75% ethanol and incubated with 5 *μ*L PI staining solution. A flow cytometer (BD FACSCalibur, San Jose, CA) was adopted to evaluate the cell cycle distribution.

Cell apoptosis was detected using FITC apoptosis detection kits (AP101, Multisciences, China). Cells were incubated with 500 *μ*L buffer, 5 *μ*L FITC-labeled Annexin V, and 10 *μ*L PI. The stained cells were evaluated using a LSRFortessa flow cytometer (BD Biosciences) [19].

### 2.11. EdU Assay

The EdU assay was performed according to the EdU cell proliferation kit (C10310, Guangzhou Ribo Biotechnology Co., Ltd., Guangzhou, China) [20]. Cells were seeded in a 24-well plate with an initial density of 1 × 10^6^ cells/mL and seeding volume of 200 *μ*L and incubated for 2 h with the culture medium containing EdU at the concentration to 10 *μ*mol/L. Next, cells were incubated with 100 *μ*L staining solution per well for 30 min in the dark at room temperature and then stained with Hoechst. Cells then were observed under a fluorescence microscope (FM-600, Shanghai Pudan Optical Instrument Co., Ltd., Shanghai, China).

### 2.12. RNA Binding Protein Immunoprecipitation (RIP) Assay

The cells were lysed with 1 mL RIPA lysis buffer (P0013B, Beyotime Biotechnology Co., Shanghai, China) in the presence of protease inhibitor in an ice bath for 1 h. The binding of circ_000829 to SRSF1 and that of SRSF1 to *SLC39A14* mRNA subtype were detected using the RIP kit (Merck Millipore, Billerica, MA). An aliquot of 10 *μ*g cell extract was collected as input. One portion of 200 *μ*g cell lysate was incubated with flag antibody, and another portion of 200 *μ*g was incubated with isotype control antibody IgG. Cell lysates were incubated with RIP buffer containing magnetic beads MA1-91878 (Thermo Fisher Scientific). The beads were added with RNase inhibitor and incubated overnight in a 4°C refrigerator. Magnetic bead products were incubated at 65°C for 45 min with 117 *μ*L RIP buffer, 15 *μ*L 10% SDS, and 18 *μ*L proteinase K. The isolated RNA was purified and stored at -80°C. The purified RNA product was used for subsequent qPCR detection. Antibodies used in this assay included rabbit anti-Flag (1 : 100, AE063, ABclonal, China) and rabbit antihuman IgG (1 : 100, ab109489, Abcam, serving as a NC). The Protein A/G magnetic beads (HY-K0202, MCE, Sovizzo Vicenza, Italia) used were 100 *μ*L/reaction, and the concentration was 1 mg/mL [21].

### 2.13. RNA Pull-Down Assay

The biotinylated circ_000829 probe was labeled by transcription *in vitro*, and 100 pmol of biotinylated circ_000829 was incubated with 200 *μ*g cytoplasmic protein extract to form an RNA-protein complex. Part of the cell extract was taken as input, and the rest was incubated with 50 *μ*L M-280 streptavidin magnetic beads (Sigma-Aldrich, St. Louis MO) precoated with RNase-free tRNA (Sigma-Aldrich) at 4°C for 3 h. Total protein was extracted to detect SRSF1 expression using western blot.

### 2.14. Xenograft Tumor Model

Specific pathogen free BALB/c nude mice (age: 6-8 weeks) were housed in separate cages with humidity of 60-65% and temperature of 22-25°C, with free access to food and water under a 12 h/12 h light-dark cycle. A total of 1 × 10^6^ A498 cells transduced either with lentivirus containing NC or circ_000829 vector were inoculated to the dorsal region of mice (*n* = 8). The mice were euthanized 30 days later and the excised tumors weighed. The tumor volumes (width × length^2^ × 0.5) were measured every 6 days (days 0, 6, 12, 18, 24, and 30).

### 2.15. Reagent Information

The reagents mainly used in the experiments are listed in Supplementary Table 3.

### 2.16. Statistical Analysis

All data were analyzed using SPSS 21.0 statistical software (IBM Corp. Armonk, NY). The measurement data are shown as mean ± standard deviation. Cell experiments were repeated in triplicate. Paired *t*-test was used for comparison between the RCC tissues and adjacent normal tissues, and unpaired *t*-test was used for comparison between the other two groups. One-way analysis of variance (ANOVA) and Tukey's post hoc test were used for data comparison among multiple groups, and repeated measures ANOVA and Bonferroni's post hoc test were used for data comparison among multiple groups at different time points. Pearson correlation was used to analyze the correlation between two factors. A *p* value < 0.05 indicated statistically significant difference.

## 3. Results

### 3.1. Overexpression of Circ_000829 Inhibits RCC Cell Proliferation while Increasing Cell Apoptosis

**Figure 1:**
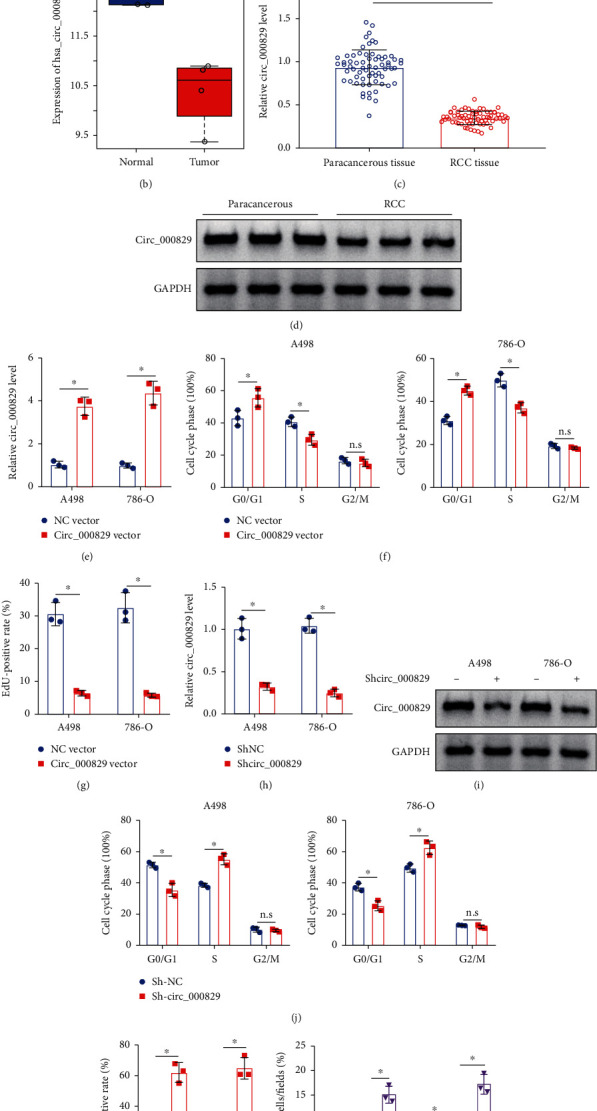
Circ_000829 is underexpressed in RCC, and its upregulation inhibited RCC cell proliferation and induced cell apoptosis. (a) Venn diagram presenting the circRNAs that may interact with SRSF1 in RCC samples predicted by intersection of the Circinteractome website and the GSE100186 dataset. (b) The hsa_circ_000829 expression in RCC samples in the GSE100186 dataset (*n* = 4 for each dataset). (c) RT-qPCR analysis of the expression of circ_000829 in clinical RCC tissues and adjacent normal tissues. (d) Northern blot analysis of circ_000829 expression in clinical RCC tissues and adjacent normal tissues (*n* = 3). (e) RT-qPCR analysis of circ_000829 overexpression efficiency in A498 and 786-O cells. (f) Cell cycle distribution detected by flow cytometry in the presence of overexpression of circ_000829. (g) Cell proliferation assessed by EdU assay in the presence of overexpression of circ_000829. (h) RT-qPCR analysis of knockdown efficiency of sh-circ_000829 in A498 and 786-O cells. (i) Knockdown efficiency of sh-circ_000829 in A498 and 786-O cells confirmed by northern blot analysis. (j) Cell cycle distribution detected by flow cytometry in the presence of sh-circ_000829. (k) Cell proliferation assessed by EdU assay in the presence of sh-circ_000829. (l) Flow cytometric analysis of apoptosis of A498 and 786-O cells in the presence of circ_000829 vector or sh-circ_000829. ^∗^*p* < 0.05. The cell experiments were conducted three times independently.

Expression of the oncoprotein SF2/SRSF1 is closely related to poor survival rate of RCC patients [7]. In order to identify the circRNAs that may regulate SRSF1, we first predicted the expression of regulatory circRNAs of SRSF1 through the bioinformatics analysis website Circinteractome. Poorly expressed circRNAs were screened in the GSE100186 dataset and Circinteractome, which yielded only hsa_circ_000829 at the intersection of potential binding circRNAs and poorly expressed circRNAs (Figure 1(a)). We then analyzed the circ_000829 expression level in the GSE100186 dataset and found that circ_000829 was downregulated in RCC samples (Figure 1(b)).

**Figure 2:**
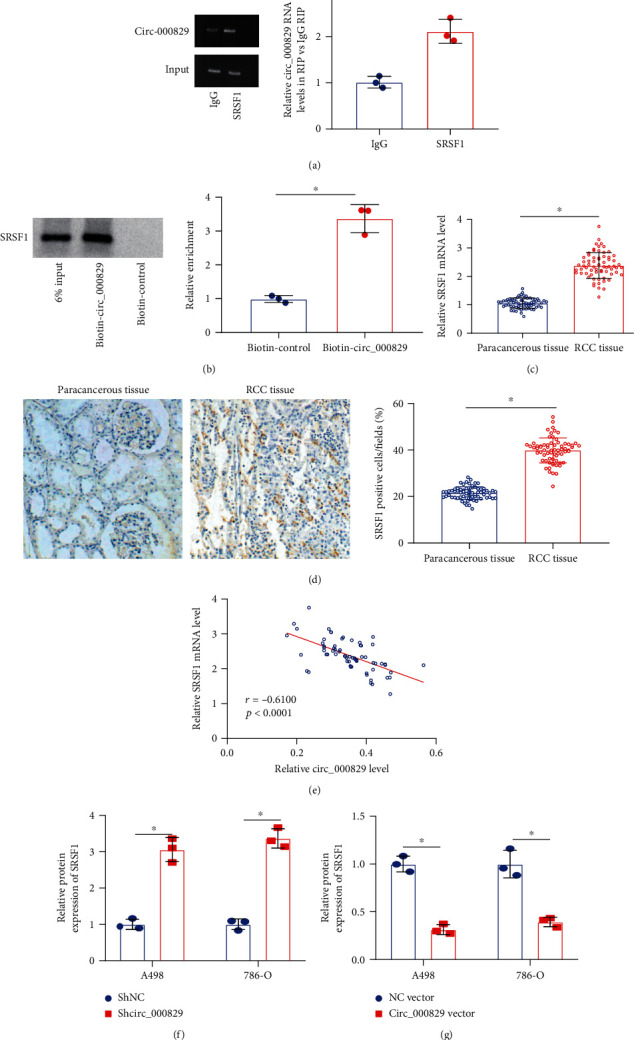
Circ_000829 binds to SRSF1 and inhibits its expression. (a) The binding of SRSF1 antibody to circ_000829 assessed by the RIP assay. (b) The binding of SRSF1 to biotin-circ_000829 in the RNA pull down assay. (c) RT-qPCR analysis of SRSF1 expression in 67 pairs of RCC and adjacent normal tissues. (d) IHC analysis of SRSF1 protein in 67 pairs of RCC and adjacent normal tissues. Blue indicates the nucleus, and brown indicates SRSF1 positive cells. (e) Pearson analysis of the correlation between the relative expression of circ_000829 and SRSF1 in RCC tissues (*n* = 67). (f) Western blot of SRSF1 expression in A498 and 786-O cells in the presence of sh-circ_000829. (g) Western blot of SRSF1 protein expression in A498 and 786-O cells in the presence of overexpression of circ_000829. ^∗^*p* < 0.05. The cell experiments were conducted three times independently.

To verify the role of circ_000829 in the development of RCC, the expression of circ_000829 was determined in clinical RCC tissues and adjacent normal tissues by RT-qPCR (Figure 1(c)) and northern blot (Figure 1(d)). It was demonstrated that the expression of circ_000829 was indeed significantly reduced in RCC tissues (Figures 1(c) and 1(d)). In addition, the overexpression efficiency of circ_000829 vector in A498 and 786-O cells was confirmed by RT-qPCR (Figure 1(e)). Moreover, overexpression of circ_000829 increased the number of G0/G1 phase-arrested cells, but decreased that of S phase-arrested cells, as demonstrated by flow cytometry (Figure 1(f) and Supplementary Figure 1A). Cell proliferation was also suppressed in the presence of circ_000829 overexpression (Figure 1(g)Figure 2A). However, transduction with sh-circ_000829 resulted in a decline in the circ_000829 expression as determined by RT-qPCR (Figure 1(h)) and northern blot (Figure 1(i)) and a reduced proportion of G0/G1 phase-arrested cells, yet enhanced cell proliferation (Figure 1(k) and Supplementary Figure 2B) and increased number of cells in the S phase (Figure 1(j) and Supplementary Figure 1B). Furthermore, knockdown of circ_000829 in A498 and 786-O cells inhibited cell apoptosis, while overexpression of circ_000829 promoted cell apoptosis (Figure 1(l)).

Thus, overexpression of circ_000829 inhibited the proliferation of RCC cells and promoted their apoptosis *in vitro*, while knockdown of circ_000829 negated the results.

### 3.2. Circ_000829 Binds to SRSF1 and Inhibits Its Expression

Initial bioinformatics analysis suggested that circ_000829 might bind to SRSF1, and we then sought to elucidate the role of circ_000829 binding to SRSF1 in RCC. RIP experiment showed that SRSF1 antibody could pull down a large amount of circ_000829 as compared to IgG antibody (Figure 2(a)). Meanwhile, when biotinylated circ_000829 was used for RNA pull down assay, the results showed a significant increase in the binding of circ_000829 to SRSF1 (Figure 2(b)), suggesting that circ_000829 could directly bind to SRSF1.

Furthermore, RT-qPCR analysis results revealed higher mRNA expression of SRSF1 in RCC tissues than in adjacent normal tissues (Figure 2(c)). Similar results were yielded for the positive expression of SRSF1 in RCC tissues by IHC staining (Figure 2(d)). Pearson analysis demonstrated that circ_000829 and SRSF1 levels were negatively correlated in RCC tissues (Figure 2(e)).

The results of western blot showed that transduction with sh-circ_000829 in A498 and 786-O cells enhanced SRSF1 expression (Figure 2(f)). However, circ_000829 overexpression reduced SRSF1 expression (Figure 2(g)).

The above results indicate that circ_000829 could bind to SRSF1 and reduce the expression of SRSF1.

### 3.3. Circ_000829 Inhibits Proliferation of RCC Cells and Promotes Their Apoptosis by Targeting SRSF1

**Figure 3:**
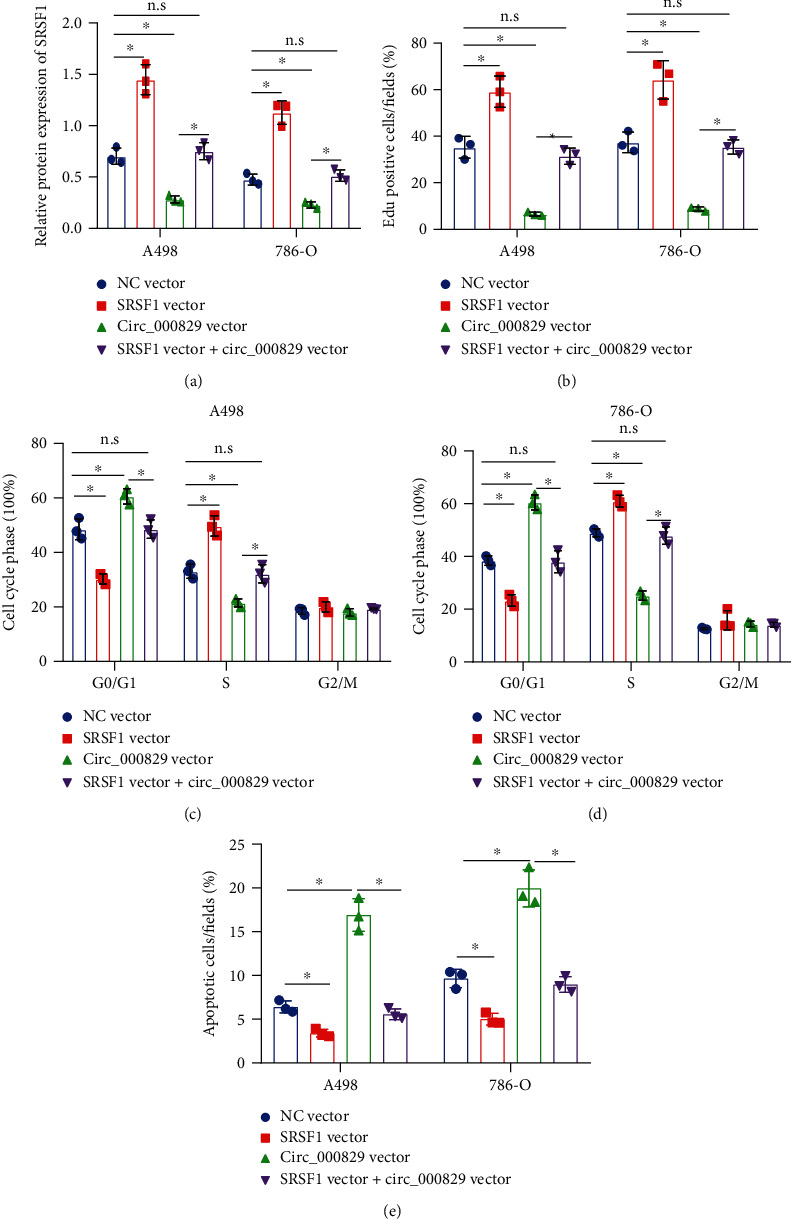
Circ_000829 targets SRSF1 to repress the proliferation of RCC cells and induce their apoptosis *in vitro*. (a) Western blot of the SRSF1 expression in A498 and 786-O cells transduced with circ_000829 vector alone or combined with SRSF1 vector. (b) Proliferation of A498 and 786-O cells transduced with circ_000829 vector alone or combined with SRSF1 vector, as assessed by EdU assay. (c) The cell cycle of A498 cells transduced with circ_000829 vector alone or combined with SRSF1 vector detected by flow cytometry. (d) The cell cycle of 786-O cells transduced with circ_000829 vector alone or combined with SRSF1 vector detected by flow cytometry. (e) Flow cytometric analysis of apoptosis of A498 and 786-O cells transduced with circ_000829 vector alone or combined with SRSF1 vector. ^∗^*p* < 0.05. n.s indicates no statistical significance. The cell experiments were conducted three times independently.

To further clarify the inhibitory mechanism of circ_000829 in A498 and 786-O cells, the two cell lines were transduced with circ_000829 vector alone or combined with SRSF1 vector. Western blot results showed that circ_000829 overexpression suppressed the SRSF1 expression (Figure 3(a)). The EdU results clearly showed that upregulation of circ_000829 markedly inhibited cell proliferation, while overexpression of SRSF1 promoted cell proliferation (Figure 3(b) and Supplementary Figure 2C). Moreover, flow cytometric data demonstrated that circ_000829 overexpression reduced the number of cells arrested in the S phase, while SRSF1 overexpression exhibited the opposite effect (Figures 3(c) and 3(d) and Supplementary Figure 1C, 1D). In addition, overexpression of circ_000829 promoted cell apoptosis, which was negated by overexpression of SRSF1. More importantly, overexpression of SRSF1 could reverse the proapoptotic effect of overexpression of circ_000829 (Figure 3(e)). Accordingly, circ_000829 repressed the proliferation of RCC cells and augmented their apoptosis by targeting SRSF1 *in vitro*.

### 3.4. SRSF1 Promotes Alternative Splicing of SLC39A14 mRNA to Produce the SLC39A14B mRNA

**Figure 4:**
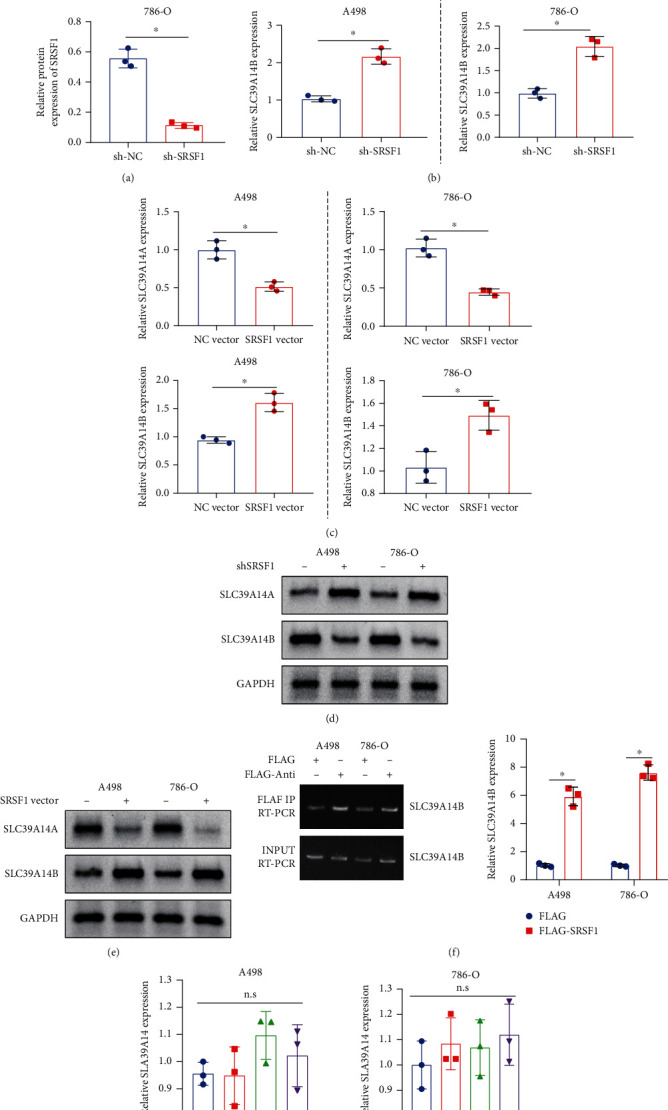
SRSF1 induces alternative splicing of SLC39A14 mRNA. (a) Knockdown efficiency of sh-SRSF1 in A498 and 786-O cells confirmed by western blot. (b) RT-qPCR analysis of the effect of sh-SRSF1 on the SLC39A14A and SLC39A14B expression. (c) RT-qPCR analysis of the effect of SRSF1 overexpression on the SLC39A14A and SLC39A14B expression. (d) SLC39A14A and SLC39A14B expression in A498 and 786-O cells following SRSF1 silencing determined by northern blot analysis. (e) SLC39A14A and SLC39A14B expressions in A498 and 786-O cells following SRSF1 overexpression determined by northern blot analysis. (f) The role of FLAG-SRSF1 in the SLC39A14A/B expression in A498 and 786-O cells assessed by RIP assay. (g) RT-qPCR analysis of SLC39A14 pre-mRNA following SRSF1 overexpression or knockdown. ^∗^*p* < 0.05. n.s indicates no statistical significance. The cell experiments were conducted three times independently.

SRSF1 has been implicated in the alternative splicing of SLC39A14 in colorectal cancer cells [7]. In order to elucidate the regulatory role of SRSF1 in SLC39A14 in RCC, we knocked down SRSF1 in A498 and 786-O cells. Western blot results confirmed the knockdown efficiency of sh-SRSF1 in A498 and 786-O cells (Figure 4(a)). The results of RT-qPCR indicated that SRSF1 knockdown caused an increase in *SLC39A14A* mRNA expression, but a decrease in *SLC39A14B* mRNA (Figure 4(b)) in A498 and 786-O cells. However, SRSF1 overexpression led to opposite effects (Figure 4(c)). Meanwhile, northern blot analysis results further validated the results of the expression changes of SLC39A14 subtypes after knockdown or overexpression of SRSF1 in A498 and 786-O cell lines (Figures 4(d) and 4(e)).

To further determine whether SRSF1 could bind to SLC39A14A and SLC39A14B, we overexpressed FLAG-tagged SRSF1 in A498 cells. RIP and RT-qPCR results showed that SRSF1 pulled down SLC39A14B (Figure 4(f)), indicating a higher affinity between SRSF1 and SLC39A14B.

Next, to verify whether SRSF1 upregulated SLC39A14 pre-mRNA expression or favored its alternate splicing to B isoform over A, we estimated the expression of SLC39A14 pre-mRNA by RT-qPCR. The results revealed that neither overexpression nor knockdown of SRSF1 had any significant effect on SLC39A14 pre-mRNA (Figure 4(g)).

Together, SRSF1 only regulated “B splicing” instead of “A splicing,” and the total level of A + B remained unchanged.

### 3.5. SLC39A14B Abrogates the Antiproliferation and Proapoptosis Effects of SLC39A14 Knockdown on RCC Cells

**Figure 5:**
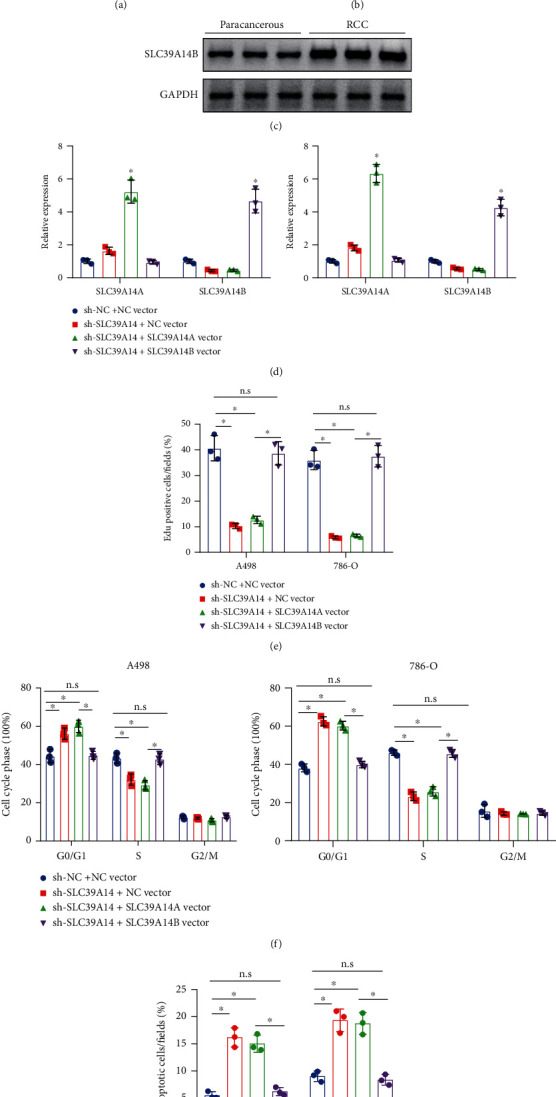
SLC39A14B abolishes the antiproliferation and proapoptosis effects of SLC39A14 knockdown on RCC cells. (a) Analysis of SLC39A14 protein expression in RCC based on GEPIA database. (b) RT-qPCR analysis of the expression of SLC39A14B in RCC tissues and adjacent normal tissues (*n* = 67). (c) Northern blot analysis of SLC39A14B expression in RCC tissues and adjacent normal tissues (*n* = 3). (d) RT-qPCR analysis of SLC39A14A and SLC39A14B expression in A498 and 786-O cells transduced with sh-SLC39A14 alone or combined with SLC39A14A vector or SLC39A14B vector. (e) The proliferation of A498 and 786-O cells transduced with sh-SLC39A14 alone or combined with SLC39A14A vector or SLC39A14B vector detected by EdU. (f) Flow cytometry detection of the cell cycle of A498 and 786-O cells transduced with sh-SLC39A14 alone or combined with SLC39A14A vector or SLC39A14B vector. (g) Flow cytometric analysis of apoptosis of A498 and 786-O cells transduced with sh-SLC39A14 alone or combined with SLC39A14A vector or SLC39A14B vector. ^∗^*p* < 0.05. n.s means no statistical significance. The cell experiments were conducted three times independently.

To clarify the effect of SLC39A14B on RCC, the expression of SLC39A14 protein in RCC samples was analyzed using the GEPIA database. The results showed that the expression of SLC39A14 in RCC samples was increased (Figure 5(a)). RT-qPCR data further confirmed that the mRNA expression of *SLC39A14B* was increased in RCC tissues compared to adjacent normal tissues (Figure 5(b)). Furthermore, northern blot analysis results revealed that *SLC39A14B* was upregulated in RCC tissues (Figure 5(c)).

SLC39A14 expression was knocked down in A498 and 786-O cells, which were then transduced with SLC39A14A vector or SLC39A14B vector. Combined transduction of sh-SLC39A14 and SLC39A14A vector increased the expression of SLC39A14A, while combined transduction of sh-SLC39A14 and SLC39A14B vector increased the expression of SLC39A14B (Figure 5(d)).

Furthermore, the EdU results displayed that the proliferation of A-498 and 786-O cells was inhibited in the SLC39A14 silencing presence, which could be reversed by overexpression of SLC39A14B but not by overexpression of SLC39A14A (Figure 5(e) and Supplementary Figure 2D). Flow cytometry detection indicated that the number of S phase-arrested cells was reduced following knockdown of SLC39A14, which could be reversed by overexpression of SLC39A14B (Figure 5(f) and Supplementary Figure 1E, 1F). In addition, overexpression of SLC39A14B abolished the proapoptotic effect of SLC39A14 knockdown in A-498 and 786-O cells (Figure 5(g)).

Therefore, overexpression of SLC39A14B can reverse the antiproliferation and proapoptosis effects of SLC39A14 knockdown on RCC cells.

### 3.6. Circ_000829 Inhibits the Tumorigenesis of RCC Cells In Vivo

**Figure 6:**
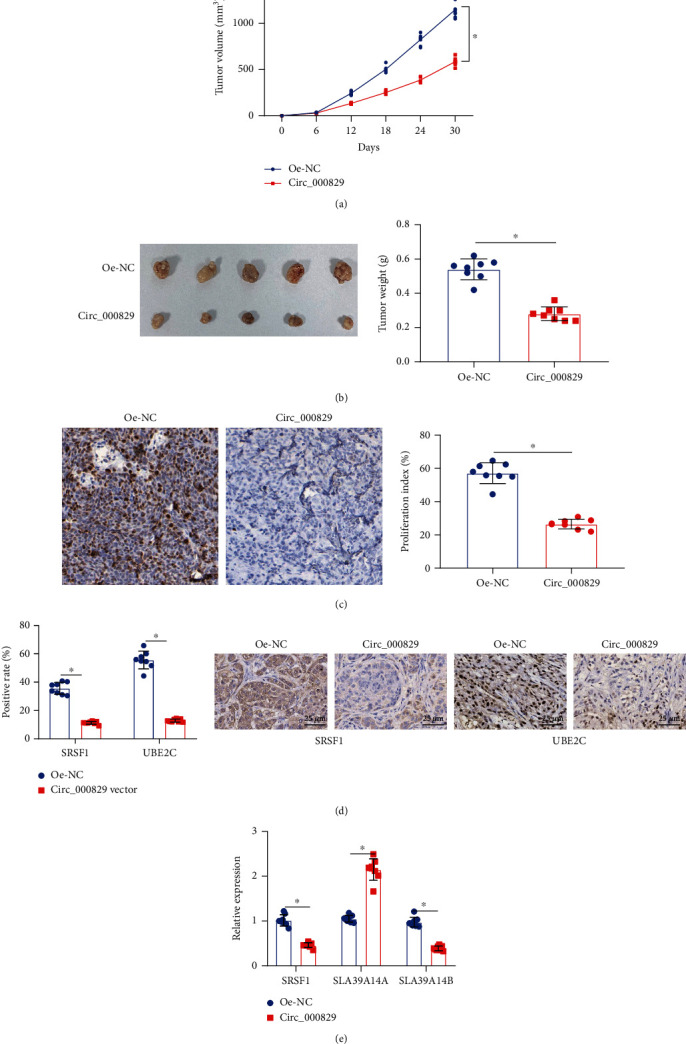
Circ_000829 suppresses the tumorigenesis of RCC cells *in vivo*. (a) Volume of the tumors at different time points after A498 cells overexpressing circ_000829 was inoculated subcutaneously in the dorsal region of nude mice. (b) Representative images of tumors excised from the nude mice euthanized at 30 days after cell inoculation and quantitation of their weight. (c) IHC analysis of the number of Ki67 positive cells after overexpression of circ_000829 in tumor tissues of nude mice. (d) IHC analysis of the expression of SRSF1 and UBE2C after overexpression of circ_000829 in tumor tissues of nude mice. (e) RT-qPCR analysis of the expression of SRSF1, SLC39A14A, and SLC39A14B in tumor tissues of nude mice after overexpression of circ_000829. *n* = 8. ^∗^*p* < 0.05.

We subcutaneously inoculated A498 cells overexpressing circ_000829 into nude mice to further verify the above findings *in vivo*. It was demonstrated that overexpression of circ_000829 reduced the tumor volume and tumor weight (Figures 6(a) and 6(b)). IHC analysis results revealed that circ_000829 overexpression reduced Ki-67 positive cells in tumor tissues, and that overexpression of circ_000829 diminished the expression of SRSF1 and UBE2C (proliferation- and invasion-related marker) in the tumor tissues (Figures 6(c) and 6(d)). Meanwhile, RT-qPCR results displayed that the expression of *SRSF1* mRNA and *SLC39A14B* mRNA was downregulated, while the expression of *SLC39A14A* mRNA was increased in tumor tissues of mice following circ_000829 overexpression (Figure 6(e)). Taken together, circ_000829 could inhibit the tumorigenesis of RCC cells in nude mice.

## 4. Discussion

Recently, circRNAs, a new type of endogenous RNAs, have been reported as regulators in many biological processes such as transcription, cell cycle control, and carcinogenesis [22, 23]. In this study, we investigated the effect of circ_000829 on RCC development. Our results showed an inhibitory role of circ_000829 in the progression of RCC by targeting SRSF1 to impede alternative splicing of SLC39A14.

We found in this study that circ_000829 was downregulated in RCC and confirmed its suppressive function in RCC. Accumulating evidence reveals that circRNAs play distinct roles as suppressors or oncogenes in various cancers [14, 24–26]. However, the role of circ_000829 in cancers has not hitherto been reported. A prior study did show that circ_001842 disrupted miRNA-502-5p-induced inhibition of SLC39A14 to exert an oncogenic effect on RCC [26], which is partially consistent with our above findings. We found that circ_000829 could bind to SRSF1 directly. SRSF1 was overexpressed in RCC cells, and circ_000829 inhibited the proliferation of RCC cells by targeting SRSF1. Martinez-Terroba et al. have proposed that SRSF1, an RNA-binding protein that has substantial effect on human pathogenesis, is implicated in splicing and functions as a proto-oncogene, and that SRSF1 overexpression enhanced the expression of oncogenic isoforms of multiple genes to promote cancer progression [27]. Moreover, as a pivotal member of serine and arginine rich protein family, SRSF1 could regulate alternative splicing of some proteins and is implicated in many biological events such as translation and senescence [28, 29]. These results partially supported our finding that circ_000829 suppressed the growth of RCC cells via targeting SRSF1.

In addition, our results indicated that SRSF1 promoted the alternative splicing of SLC39A14 to SLC39A14B, which contributed to the proliferation of RCC cells. Consistently, the study of Thorsen et al. has suggested that SRSF1 was involved in alternative splicing of SLC39A14 to drive the proliferation of colorectal cancer cells [7]. Alternative splicing is considered an important factor related to physiological complexity, which occurs in 95% or more of human multiexon genes [30]. However, alternative splicing usually occurs in the exons and introns of circRNA, making it difficult to determine its internal structure; therefore, circRNA splicing may be cancer-specific [31]. The data of Song et al. have also reported that changes in splicing patterns could alter the function of proteins and showed that other splicing events could be used as promising prognostic biomarkers for clear cell RCC [32]. Therefore, we suppose that SRSF1-mediated alternative splicing may play an important role in RCC. In addition, as a glycosylated protein located on the plasma membrane, SLC39A14 acts as a metal ion transporter for iron, zinc, manganese, and cadmium [8, 33]. SLC39A14, also known as ZIP14, has two different isoforms, namely, SLC39A14A (ZIP14A) and SLC39A14B (ZIP14B), of which the latter one has a higher affinity for Cd^2+^ than does SLC39A14A [8]. The affinity difference between SLC39A14A and SLC39A14B for Cd^2+^ is a result of alternative splicing [10]. Cd^2+^ can promote cell migration and invasiveness and is reported to promote the process of RCC [34], indicating that SLC39A14B is a promoter of RCC. In short, SRSF1 promoted the alternative splicing of SLC39A14 into SLC39A14B, which aggravated RCC. We also performed *in vivo* experiments in mice, which revealed that the overexpression of circ_000829 not only downregulated the expression of SRSF1 and SLC39A14B but also reduced the volume and weight of the tumor, all of which further confirmed the above findings.

## 5. Conclusion

**Figure 7:**
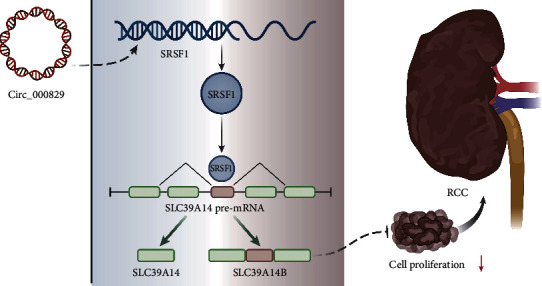
Mechanistic graph summarizing a possible regulatory role of circ_000829 in RCC. Circ_000829 inhibits the alternative splicing of *SLC39A14* mRNA by binding to SRSF1, thereby inhibiting the proliferation of RCC cells *in vitro* and tumorigenesis *in vivo*.

In summary, circ_000829 had antitumor effects in RCC. Circ_000829 suppressed the alternative splicing of SLC39A14 by targeting SRSF1, thereby impeding the progression of RCC (Figure 7), which may offer a new therapeutic biomarker or target for RCC. However, this study is still limited by the lack of detection on the concentration of metal ions in clinical samples and cell function experiments. As a metal transporter, how SLC39A14 is affected by the concentration of metal ions in this study remains unclear and needs future investigations.

## Data Availability

The datasets generated and/or analyzed during the current study are available from the corresponding author on reasonable request.

## Abbreviations

SRSF1: Serine and arginine rich splicing factor 1
ANOVA: Analysis of variance
BSA: Bovine serum albumin
circRNAs: Circular RNAs
EdU: 5-ethynyl-2′-deoxyuridine
FBS: Fetal bovine serum
FITC: Fluorescein isothiocyanate
IHC: Immunohistochemistry
HRP: Horseradish peroxidase
WHO: World Health Organization
ISUP: International Society of Urological Pathology
MEM: Minimum essential medium
NC: Negative control
oe-: Overexpression
PBS: Phosphate buffer saline
PI: Propidium iodide
RCC: Renal cell carcinoma
RIP: RNA binding protein immunoprecipitation
RIPA: Radioimmunoprecipitation assay
RPMI: Roswell Park Memorial Institute
RT-qPCR: Reverse transcription-quantitative polymerase chain reaction
sh-: Short hairpin RNA.

## Supplementary Materials

Supplementary Materials Supplementary Figure 1: representative images of cell cycle distribution by flow cytometry. Supplementary Figure 2: representative images of cell proliferation by flow cytometry. Supplementary Table 1: descriptive clinical characteristics of patients with RCC. Supplementary Table 2: primer sequences for RT-qPCR. Supplementary Table 3: reagent information list.

## References

[1] Moch H. (2013). An overview of renal cell cancer: pathology and genetics. *Seminars in Cancer Biology*.

[2] Diaz-Montero C. M., Rini B. I., Finke J. H. (2020). The immunology of renal cell carcinoma. *Nature Reviews. Nephrology*.

[3] Atkins M. B., Tannir N. M. (2018). Current and emerging therapies for first-line treatment of metastatic clear cell renal cell carcinoma. *Cancer Treatment Reviews*.

[4] Bui T. O., Dao V. T., Nguyen V. T., Feugeas J. P., Pamoukdjian F., Bousquet G. (2022). Genomics of clear-cell renal cell carcinoma: a systematic review and meta- analysis. *European Urology*.

[5] Jeong S. (2017). SR proteins: binders, regulators, and connectors of RNA. *Molecules and Cells*.

[6] Zhang J., Harvey S. E., Cheng C. (2019). A high-throughput screen identifies small molecule modulators of alternative splicing by targeting RNA G-quadruplexes. *Nucleic Acids Research*.

[7] Thorsen K., Mansilla F., Schepeler T. (2011). Alternative splicing of SLC39A14 in colorectal cancer is regulated by the Wnt pathway. *Molecular & Cellular Proteomics*.

[8] Aydemir T. B., Cousins R. J. (2018). The multiple faces of the metal transporter ZIP14 (SLC39A14). *The Journal of Nutrition*.

[9] Chen C., Xue S., Zhang J. (2017). DNA-methylation-mediated repression of miR-766-3p promotes cell proliferation via targeting SF2 expression in renal cell carcinoma. *International Journal of Cancer*.

[10] Pan X. W., Xu D., Chen W. J. (2021). USP39 promotes malignant proliferation and angiogenesis of renal cell carcinoma by inhibiting VEGF-A165b alternative splicing via regulating SRSF1 and SRPK1. *Cancer Cell International*.

[11] Tang X., Ren H., Guo M., Qian J., Yang Y., Gu C. (2021). Review on circular RNAs and new insights into their roles in cancer. *Computational and Structural Biotechnology Journal*.

[12] Jin J., Sun H., Shi C. (2020). Circular RNA in renal diseases. *Journal of Cellular and Molecular Medicine*.

[13] Chen Q., Liu T., Bao Y. (2020). CircRNA cRAPGEF5 inhibits the growth and metastasis of renal cell carcinoma via the miR-27a-3p/TXNIP pathway. *Cancer Letters*.

[14] Zhang D., Yang X. J., Luo Q. D. (2019). Down-regulation of circular RNA_000926 attenuates renal cell carcinoma progression through miRNA-411-dependent CDH2 inhibition. *The American Journal of Pathology*.

[15] Yu Y., Fang L. (2022). _CircRPAP2_ regulates the alternative splicing of PTK2 by binding to SRSF1 in breast cancer. *Cell Death Discov*.

[16] Han D., Yu Y., Yu N. (2020). Prediction models for clear cell renal cell carcinoma ISUP/WHO grade: comparison between CT radiomics and conventional contrast-enhanced CT. *The British Journal of Radiology*.

[17] Schneider T., Schreiner S., Preusser C., Bindereif A., Rossbach O. (2018). Northern blot analysis of circular RNAs. *Methods in Molecular Biology*.

[18] Liu T., Shi Q., Yang L. (2021). Long non-coding RNAs HERH-1 and HERH-4 facilitate cyclin A2 expression and accelerate cell cycle progression in advanced hepatocellular carcinoma. *BMC Cancer*.

[19] Pan W., Wang L., Zhang X. F. (2019). Hypoxia-induced microRNA-191 contributes to hepatic ischemia/reperfusion injury through the ZONAB/cyclin D1 axis. *Cell Death and Differentiation*.

[20] Zhu J., Liu B., Wang Z. (2019). Exosomes from nicotine-stimulated macrophages accelerate atherosclerosis through miR-21-3p/PTEN-mediated VSMC migration and proliferation. *Theranostics*.

[21] Spitzer J., Hafner M., Landthaler M. (2014). PAR-CLIP (photoactivatable ribonucleoside-enhanced crosslinking and immunoprecipitation): a step-by-step protocol to the transcriptome-wide identification of binding sites of RNA-binding proteins. *Methods in Enzymology*.

[22] Wang S., Xia P., Zhang L. (2019). Systematical identification of breast cancer-related circular RNA modules for deciphering circRNA functions based on the non-negative matrix factorization algorithm. *International Journal of Molecular Sciences*.

[23] Yang L., Zou X., Zou J., Zhang G. (2021). Functions of circular RNAs in bladder, prostate and renal cell cancer (review). *Molecular Medicine Reports*.

[24] Ouyang J., Long Z., Li G. (2020). Circular RNAs in gastric cancer: potential biomarkers and therapeutic targets. *BioMed Research International*.

[25] Li J., Huang C., Zou Y., Ye J., Yu J., Gui Y. (2020). CircTLK1 promotes the proliferation and metastasis of renal cell carcinoma by sponging miR-136-5p. *Molecular Cancer*.

[26] Zeng J., Feng Q., Wang Y. (2020). Circular RNA circ_001842 plays an oncogenic role in renal cell carcinoma by disrupting microRNA-502-5p-mediated inhibition of SLC39A14. *Journal of Cellular and Molecular Medicine*.

[27] Martínez-Terroba E., Ezponda T., Bértolo C. (2018). The oncogenic RNA-binding protein SRSF1 regulates LIG1 in non-small cell lung cancer. *Laboratory Investigation*.

[28] Paz S., Ritchie A., Mauer C., Caputi M. (2021). The RNA binding protein SRSF1 is a master switch of gene expression and regulation in the immune system. *Cytokine & Growth Factor Reviews*.

[29] Jaiswal A., Singh A. K., Tamrakar A., Kodgire P. (2021). Unfolding the role of splicing factors and RNA debranching in AID mediated antibody diversification. *International Reviews of Immunology*.

[30] Jiang W., Chen L. (2021). Alternative splicing: human disease and quantitative analysis from high- throughput sequencing. *Computational and Structural Biotechnology Journal*.

[31] Feng J., Chen K., Dong X. (2019). Genome-wide identification of cancer-specific alternative splicing in circRNA. *Molecular Cancer*.

[32] Song J., Liu Y. D., Su J., Yuan D., Sun F., Zhu J. (2019). Systematic analysis of alternative splicing signature unveils prognostic predictor for kidney renal clear cell carcinoma. *Journal of Cellular Physiology*.

[33] Tinkov A. A., Paoliello M., Mazilina A. N. (2021). Molecular targets of manganese-induced neurotoxicity: a five-year update. *International Journal of Molecular Sciences*.

[34] Akin R., Hannibal D., Loida M., Stevens E. M., Grunz-Borgmann E. A., Parrish A. R. (2019). Cadmium and lead decrease cell-cell aggregation and increase migration and invasion in Renca mouse renal cell carcinoma cells. *International Journal of Molecular Sciences*.

