# Supplementary material for: Circ_000829 Plays an Anticancer Role in Renal Cell Carcinoma by Suppressing SRSF1-Mediated Alternative Splicing of SLC39A14
Source: Oxid Med Cell Longev. 2022 Aug 26;2022:8645830. doi: 10.1155/2022/8645830 (PMC9439915; doi:10.1155/2022/8645830)
Supplement: Supplementary Materials — Supplementary Figure 1: representative images of cell cycle distribution by flow cytometry. Supplementary Figure 2: representative images of cell proliferation by flow cytometry. Supplementary Table 1: descriptive clinical characteristics of patients with RCC. Supplementary Table 2: primer sequences for RT-qPCR. Supplementary Table 3: reagent information list. [file 8645830.f1.zip › 8645830.f1/Supplementary Tables (1).docx]

**Supplementary Table 1.** Descriptive clinical characteristics of patients with RCC

| Variables | No. of patients (%) | Variables | No. of patients (%) |
| --- | --- | --- | --- |
| Age at surgery (years) |  | KPS |  |
| < 60 | 36 | ≥ 80 | 52 |
| ≥ 60 | 31 | < 80 | 15 |
| Gender |  | Lymph node involvement |  |
| Male | 43 | N0 | 41 |
| Female | 24 | N1 | 26 |
| BMI (kg/m2) | 22.45 ± 2.03 | Pathological grade |  |
| < 24 | 56 | I-II | 40 |
| ≥ 24 | 11 | III | 37 |
| Pathology |  | T stage |  |
| Clear cell | 53 | T1-T2 | 39 |
| Papillar | 6 | T3-T4 | 28 |
| Chromophobe | 5 | SCr (μmol/L) | 81.3 ± 14.8 |
| Others | 3 | < 82.1(F)/97.0(M) | 17/35 |
| Comorbidities (HP/DM) |  | ≥ 82.1(F)/97.0(M) | 44750 |
| No | 35 | CysC (mg/L) | 1.05 ± 0.39 |
| HP | 12 | < 1.09 | 40 |
| DM | 15 | ≥ 1.09 | 27 |
| HP + DM | 5 | eGFR (mL/min/1.73m2) | 81.3 ± 24.0 |
|  |  | ≥ 60 | 54 |
|  |  | < 60 | 13 |

Note: BMI, body mass index; HP, hypertension; DM, diabetes mellitus; KPS, Karnofsky performance status; CysC, cystatin C; SCr, serum creatinine; eGFR, estimated glomerular filtration rate. It is calculated by CKD-EPI creatinine–cystatin C equation (2012), eGFR =135 × min (SCr / κ,1)^α^ × max (SCr / κ,1) ^-0.601^ × min (CysC / 0.8, 1) ^-0.375^ × max (CysC / 0.8, 1) ^-0.711^ × 0.995 ^Age^.

**Supplementary Table 2.** Primer sequences for RT-qPCR

| Gene | Sequences |
| --- | --- |
| SLC39A14A | Forward: 5’-TTTCTCAGTGTCTCACTGATTAA-3’ |
|  | Reverse: 5’-GAATAGCGCGTTAGACAGC-3’ |
| SLC39A14B | Forward: 5’-TGTTGAAGTGTGGGGATACGGT-3’ |
|  | Reverse: 5’-ATGAAGTAGAGCAGCAGCCTCT-3’ |
| Circ_000829 | Forward: 5’- AATTCTCAACAACAGCAGCTC-3’ |
|  | Reverse: 5’- TGGTCCATCTTTGCTGGATTC-3’ |
| β-actin | Forward: 5’- AGCCTTCCTTCCTGGGCATGG -3’ |
|  | Reverse: 5’- TGTGTTGGCGTACAGGTCTTTG -3’ |
| SRSF1 | Forward: 5’-TCAGGCAAGGTTGTCCAAGT-3’ |
|  | Reverse: 5’-GTAACTGCGACTCCTGCTGT-3’ |

Note: RT-qPCR, reverse transcription-quantitative polymerase chain reaction.

**Supplementary Table 3.** Reagent information list

| Reagent name | Catalog number | Vendors/locations |
| --- | --- | --- |
| NorthernMax Kit | AM1940 | ThermoFisher, USA |
| Normal goat serum | C-0005 | Haoran, China |
| SRSF1 antibody | ab38017 | Abcam, UK |
| Goat anti-rabbit IgG | ab6785 | Abcam, UK |
| Horseradish peroxidase-labeled streptavidin protein working solution | 0343-10000U | Imunbio, China |
| 3,3'-diaminobenzidine tetrahydrochloride | ST033 | Whiga, China |
| Hematoxylin | PT001 | Bogoo, China |
| A498 cell line | 3111C0001CCC000171 | Resource Center, Institute of Basic Medical Sciences, Academy of Medical Sciences, China |
| 786-O cell line | 111C0001CCC000243 | Resource Center, Institute of Basic Medical Sciences, Academy of Medical Sciences, China |
| Lipofectamine 2000 | 11668030 | Invitrogen, USA |
| TRIzol | 15596-018 | Solarbio, China |
| Reverse Transcription Kit | 5081963001 | Roche, Germany |
| SYBR Green qPCR Master Mix | B21202 | Bimake, China |
| High-efficiency radioimmunoprecipitation assay lysis buffer | R0010 | Solarbio, China |
| SLC39A14 antibody | ab106568 | Abcam, UK |
| SRSF1 antibody | ab38017 | Abcam, UK |
| Ki67 antibody | ab15580 | Abcam, UK |
| UBE2C antibody | ab252940 | Abcam, UK |
| β-actin antibody | ab8226 | Abcam, UK |
| Goat anti-rabbit IgG | ab205718 | Abcam, UK |
| Goat anti-mouse IgG | ab6789 | Abcam, UK |
| Annexin-FITC apoptosis detection kit | AP101 | Multisciences, China |
| EdU Cell Proliferation Kit | C10310 | Ribo, China |
| RIP Kit | 17-700 | Merck Millipore, USA |
| Magnetic bead | MA1-91878 | ThermoFisher, USA |
| Streptavidin magnetic bead | M-280 | Sigma, USA |
