# Supplementary figures and images for: Circ_000829 Plays an Anticancer Role in Renal Cell Carcinoma by Suppressing SRSF1-Mediated Alternative Splicing of SLC39A14
Source: Oxid Med Cell Longev. 2022 Aug 26;2022:8645830. doi: 10.1155/2022/8645830 (PMC9439915; doi:10.1155/2022/8645830)

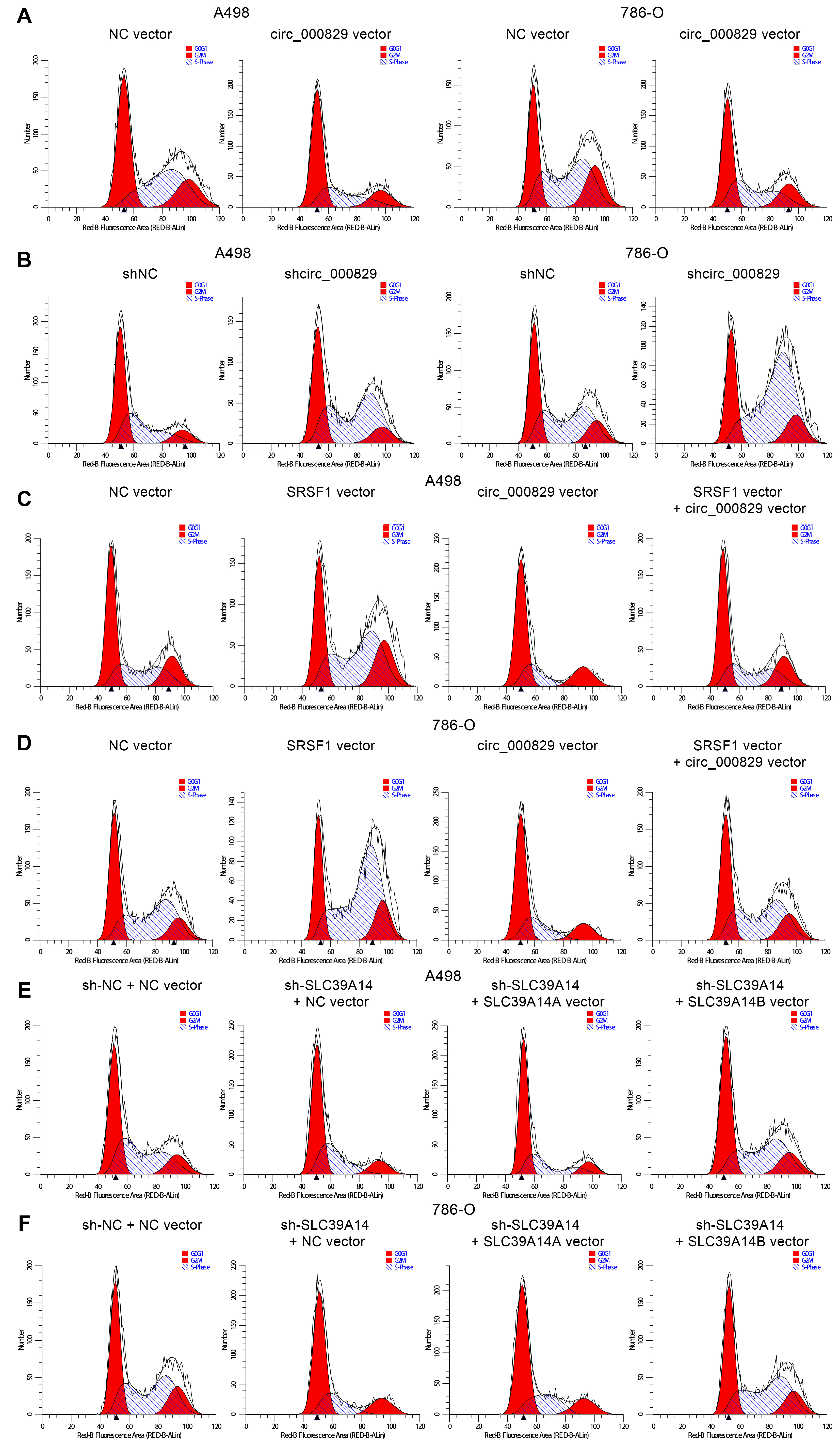

Supplement: Supplementary Materials — Supplementary Figure 1: representative images of cell cycle distribution by flow cytometry. Supplementary Figure 2: representative images of cell proliferation by flow cytometry. Supplementary Table 1: descriptive clinical characteristics of patients with RCC. Supplementary Table 2: primer sequences for RT-qPCR. Supplementary Table 3: reagent information list. [file 8645830.f1.zip › 8645830.f1/Figure S1 (1).jpg]

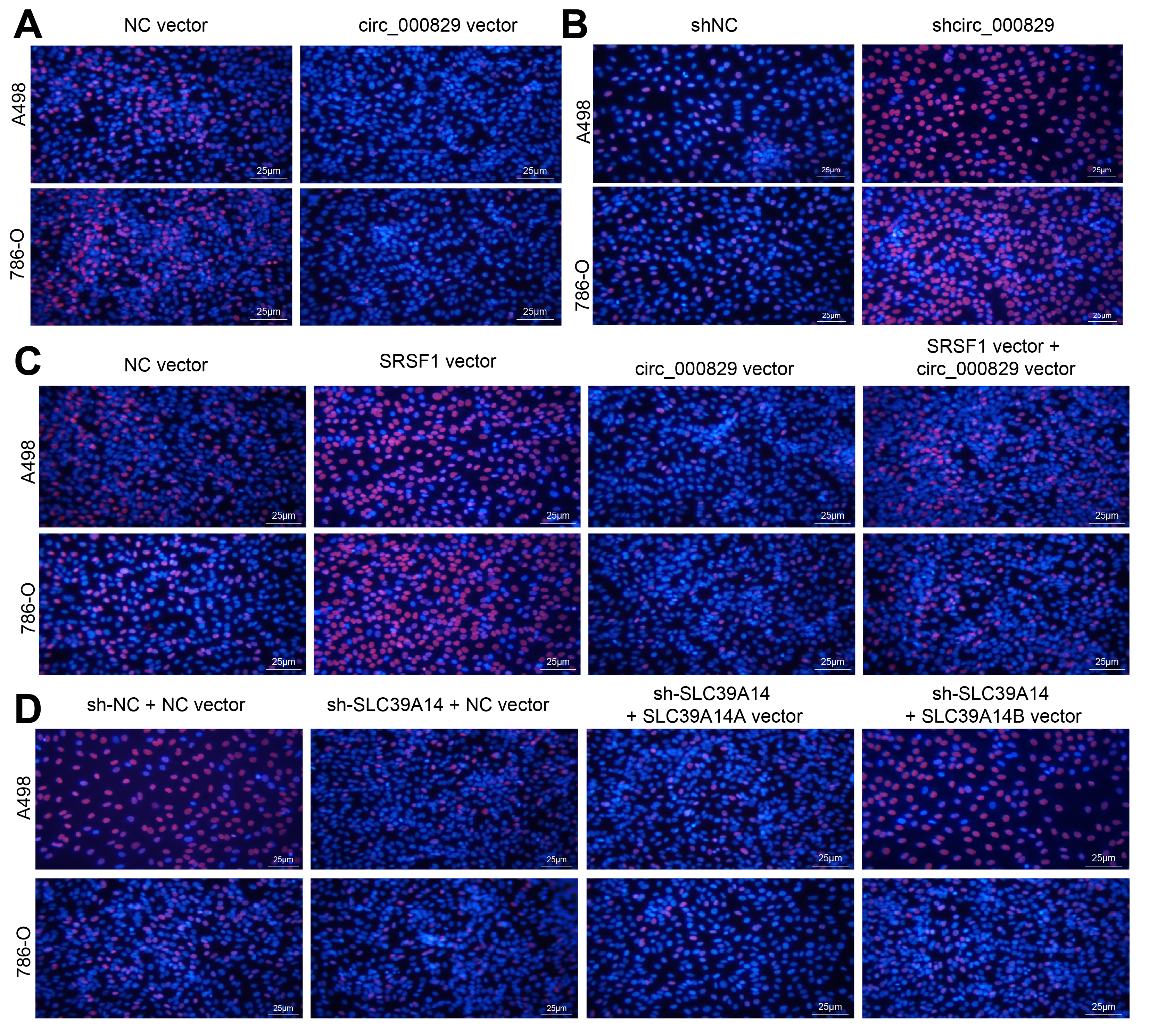

Supplement: Supplementary Materials — Supplementary Figure 1: representative images of cell cycle distribution by flow cytometry. Supplementary Figure 2: representative images of cell proliferation by flow cytometry. Supplementary Table 1: descriptive clinical characteristics of patients with RCC. Supplementary Table 2: primer sequences for RT-qPCR. Supplementary Table 3: reagent information list. [file 8645830.f1.zip › 8645830.f1/Figure S2 (1).jpg]
